# Development and Validation of an Autophagy-Related Gene Signature for Predicting the Prognosis of Hepatocellular Carcinoma

**DOI:** 10.1155/2021/7771037

**Published:** 2021-10-28

**Authors:** Jianlin Zhang, Min Zhang, Jin Huang, Gaosong Zhang, Chong Li, Xingyu Wang, Weihao Kong

**Affiliations:** ^1^Department of Emergency Surgery, Department of Emergency Medicine, The First Affiliated Hospital of Anhui Medical University, Hefei, Anhui, China; ^2^Department of Anesthesiology, Jiulongpo People's Hospital, Chongqing, China; ^3^Department of Pathology, The Second People's Hospital of Hefei, Hefei, Anhui, China; ^4^Department of Ultrasound, The First Affiliated Hospital of Anhui Medical University, Hefei, Anhui, China

## Abstract

**Purpose:**

Autophagy is a lysosomal degradation pathway that is essential for maintaining the homeostasis of the intracellular environment. Mounting evidence indicates that autophagy plays an essential role in the occurrence and development of hepatocellular cancer (HCC). This research is aimed at exploring the prognostic value of autophagy-related genes (ARGs) in HCC patients.

**Methods:**

The Wilcoxon test was used to identify differentially expressed ARGs in The Cancer Genome Atlas (TCGA) HCC cohort. Then, the TCGA cohort was randomly divided into training and testing groups. Cox and LASSO regression models were used to screen for autophagy-related genes that affect overall survival (OS) in the TCGA training group. Based on the coefficient of risk genes, we constructed an autophagy-related gene signature for predicting the prognosis of HCC patients. Finally, we validated the prognostic significance of autophagy-related gene signature using the TCGA testing group and three external datasets.

**Results:**

ATG10, BIRC5, GAPDH, and TMEM74 are risk genes for OS. According to the optimal cutoff value of risk score in each HCC dataset, HCC patients can divide into high- and low-risk groups. ARG risk score can significantly distinguish HCC patients with different survival outcomes. Meanwhile, the ARG risk score is independently correlated with OS in multiple HCC cohorts.

**Conclusions:**

The autophagy-related risk score can effectively screen high-risk HCC patients and provide guidance for clinical prevention and treatment of HCC.

## 1. Introduction

Hepatocellular carcinoma (HCC) is the third leading cause of cancer-related death in China and the fourth leading cause of cancer-related death in the world. Despite significant advances in the diagnosis and treatment of HCC in recent years, the prognosis of hepatocellular carcinoma is still poor, owing to its high invasiveness [1, 2]. Therefore, it is essential to explore the molecular mechanism of the occurrence and development of liver cancer, as it can lead to a new treatment strategy for the prevention and treatment of hepatocellular carcinoma.

Autophagy refers to the process of using lysosomes to degrade self-damaged organelles and macromolecules under the regulation of autophagy-related genes, thereby maintaining the needs of the cells themselves and the renewal of organelles. Changes in the level of autophagy are associated with a variety of human diseases, such as cancer, autoimmune diseases, and central nervous system diseases [3–6]. Several studies have shown that autophagy can inhibit the growth and invasion of tumor cells in the early stages of many tumors. However, in the advanced tumor stages, autophagy can promote the rapid growth of tumor cells by degrading aging and damaged organelles and macromolecules, thereby promoting the malignant transformation of tumor cells [4, 6]. The change in autophagy level is closely related to the development of liver cancer. The main types of autophagy in the progression of liver cancer are mainly molecular chaperone-mediated autophagy (CMA) and macroautophagy [7]. On the one hand, mice with weakened CMA can increase the vulnerability to oxidative stress, worsen liver function, and accelerate metabolic abnormalities, thereby promoting the occurrence of hepatic adenoma [8]. When it is progressing to a malignant tumor, the tumor cells show a significant increase in CMA activity in order to maintain the metabolic shift of cancerous cells [9]. On the other hand, macroautophagy plays an antitumor role in the progression of liver cancer. The reduction in autophagy level is associated with malignant transformation and poor prognosis of liver cancer. In liver cancer, a decrease in the level of autophagy can cause the appearance of the autophagy protein p62, and p62 can promote the release of nuclear factor erythroid 2-related factor 2 (Nrf2), which in turn encourages the progression of liver fibrosis and liver cancer [10, 11]. Therefore, autophagy as a target for antitumor may have important clinical significance in the future. Since autophagy plays an essential role in the occurrence and development of cancer, it is of great clinical importance to find autophagy-related tumor biomarkers.

With the popularization of high-throughput sequencing technology in recent years, it is feasible to explore the relationship between autophagy-related genes (ARGs) and the prognosis of patients with hepatocellular carcinoma. Therefore, in our study, we systematically analyzed the differentially expressed ARGs in HCC by using the TCGA database and selected differentially expressed ARGs significantly associated with OS in the TCGA training group. Based on the cutoff value of risk score in the TCGA training group, HCC patients could be divided into high- and low-risk groups. Finally, we explored the prognostic role of the ARG risk score in the TCGA training group, TCGA testing group, and three external datasets.

## 2. Material and Methods

### 2.1. Data Acquisition

The Human Autophagy Database (http://autophagy.lu/clustering/index.html) is a specialized database for preserving genes associated with human autophagy. We acquired the gene symbols of 232 autophagy-related genes from the database. Then, we extracted the expression matrix of autophagy-related genes from 374 hepatocellular carcinoma patients in the TCGA database (https://portal.gdc.cancer.gov/) and obtained the clinicopathological features and prognostic information of HCC patients from the cBioPortal online website (https://www.cbioportal.org/). Differentially expressed ARGs between tumor samples and normal samples were determined by the Wilcoxon test. The criteria for screening differential genes were ∣log_2_FC | >1 and FDR < 0.05. Normalization was performed by converting the expression matrix of the autophagy-related genes using the formula log_2_(*x* + 1) . The staging of HCC patients was determined by using the 7th edition of AJCC (American Joint Committee on Cancer) staging.

### 2.2. Functional Enrichment Analysis of ARGs

Gene ontology (GO) is a database for the definition and description of genes and protein functions for a variety of species. GO annotations include molecular function (MF), biological process (BP), and cellular components (CC). Through these three functional categories, the function of a gene can be deeply described. Kyoto Encyclopedia of Genes and Genomes (KEGG) is a comprehensive database that integrates information on genomic, chemical, and system functions. Based on this database, we can further speculate on ARG-related signaling pathways. Then, we obtained the enrichment function and pathways of differentially expressed ARGs through the cluster profile package in R software.

### 2.3. Establishment and Validation of Autophagy-Related Gene Signature for OS

We matched the prognostic information of HCC patients with the liver cancer samples and finally obtained 367 HCC patients with prognostic information. A good predictive model should have internal and external validation, so we randomly divided the TCGA cohort into TCGA training (*n* = 183) and TCGA testing groups (*n* = 184) using the “sample” function in R language. Then, univariate regression analysis was used to explore differentially expressed ARGs that were significantly associated with the OS in the TCGA training group. The LASSO regression model was used to reduce the false-positive result caused by model overfitting. Furthermore, we constructed the OS gene signature by incorporating variables from multivariate regression analysis using the stepwise method. Finally, we validated the prognostic role of risk scores in the TCGA testing group, and three external datasets.

### 2.4. ARG Risk Score Computation

The ARG risk score of each HCC patient was computed by the expression value of the risk gene screened by multivariate regression analysis and the corresponding regression coefficient. The risk score for HCC patients is calculated as follows: risk score = 0.556∗ATG10 + 0.217∗BIRC5 + 0.403∗GAPDH + 0.765∗TMEM74 for OS. Relative operating characteristic (ROC) analysis is used to determine the optimal cutoff value in each HCC dataset. Based on the optimal risk score of each dataset, we divided HCC patients into high-risk and low-risk groups. The Kaplan-Meier curve was used to map the survival time of patients in high- and low-risk groups, and the log-rank test was conducted to compare the significance of high- and low-risk groups.

### 2.5. RNA Extraction and qRT-PCR

We retrospectively analyzed the prognosis of 60 HCC patients who underwent liver resection at the First Affiliated Hospital of Anhui Medical University (AHMU) from 2010 to 2013. This study was authorized by the Ethics Committee of the First Affiliated Hospital of Anhui Medical University, and all patients signed informed consent. All HCC tumor tissues are stored in the refrigerator at -80 degrees. According to the instructions of the reagents, we used TRIzol (Invitrogen) to extract total RNA from tumor tissues and used the total RNA for reverse transcription to obtain cDNA (Takara Bio Inc.). Finally, we used SYBR Green reagent (Thermo Fisher) for real-time quantitative PCR. For qRT-PCR, each sample was repeated in triplicate. U6 serves as a reference standard for the quantification of each gene. The relative expression level of each gene was calculated using 2–*ΔΔ*CT. The qRT-PCR was carried out with the Bio-Rad PCR thermocycling Instrument. The primer sequences of these genes are shown in Supplementary Table 2.

### 2.6. Statistical Analysis

Statistical analyses were analyzed using SPSS 24.0 (Chicago, IL, USA) and R 3.5.1 software (https://www.r-project.org/). We used R packages, including limma, pheatmap, ggpubr, clusterProfiler, survival, survminer, and survivalROC. The categorical variable is represented by frequency; if the continuous variable satisfies the normal distribution, it is expressed by mean ± standard deviation; if it does not meet the normal distribution, it is expressed by median (interquartile range). Comparisons between categorical variables were utilized using chi-square analysis, and comparisons between continuous variables were performed using the Kruskal-Wallis test. Autophagy-related risk genes were identified using the least absolute shrinkage and selection operator (LSAAO) and Cox regression model. Time-dependent ROC curves were used to analyze the performance of autophagy-related gene signature to predict overall survival. A *P* value of below 0.05 was considered statistically significant.

## 3. Results

### 3.1. Clinicopathological Parameters of the Included Cohort

We randomly divided the entire TCGA dataset into training and testing groups, of which 183 were in the training group, and 184 were in the testing group. The entire dataset had 248 men and 119 women. The patients of AJCC stage I, II, III, and IV were 171, 85, 83, and 4, respectively. There were no significant differences in age, gender, AJCC staging, differentiation, survival status, and survival time between the training group and the testing group (Table 1). Meanwhile, the clinicopathological characteristics of the ICGC, GSE116174, and the AHMU dataset are shown in Supplementary Table 1.

### 3.2. Identification of Differentially Expressed Autophagy-Correlated Genes

**Figure 1:**
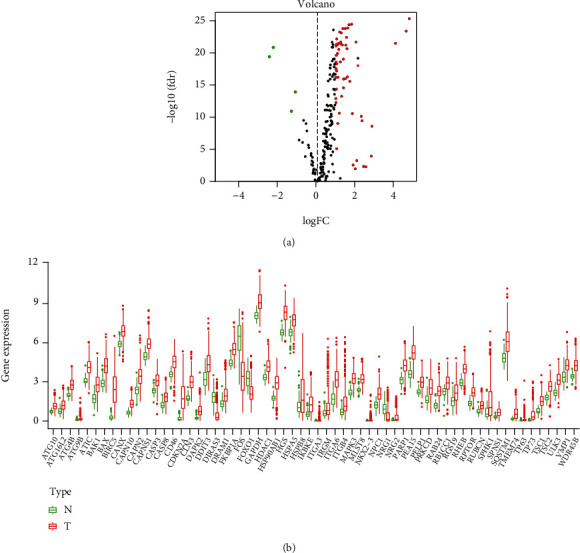
Differentially expressed analysis of autophagy-correlated genes in the TCGA HCC cohort. (a) Volcano plot of the autophagy-related genes. Notes: TCGA: The Cancer Genome Atlas; HCC: hepatocellular carcinoma; red: upregulated genes; green: downregulated genes; black: nondifferentially expressed genes. (b) Expression of 62 differentially expressed autophagy-related genes in HCC patients and normal controls. Notes: HCC: hepatocellular carcinoma; red represents tumor samples, and the green represents normal controls.

We used the Wilcoxon test to analyze the expression matrix of tumor samples and normal samples. Based on the screening criteria (∣log_2_FC | >1 and FDR < 0.05), we obtained a total of 62 differentially expressed ARGs, of which 58 were upregulated, and 4 were downregulated. Volcano map of differentially expressed autophagy-related genes is shown in Figure 1(a). Supplementary Figure 1 is a cluster heat map showing the expression matrix of differentially expressed autophagy-related genes, and Figure 1(b) is a box plot showing the expression levels of differentially expressed autophagy-related genes in tumor samples and normal samples. Among these differentially expressed ARGs, BIRC5 has the highest fold difference (log_2_FC = 4.8), and its FDR value is 4.82*E* − 26. At the same time, most of the differentially expressed ARGs are significantly increased in HCC tissues, thus revealing that they play an indispensable role in promoting the occurrence and development of liver cancer.

### 3.3. Functional Annotation of the Differentially Expressed Autophagy-Correlated Genes

**Figure 2:**
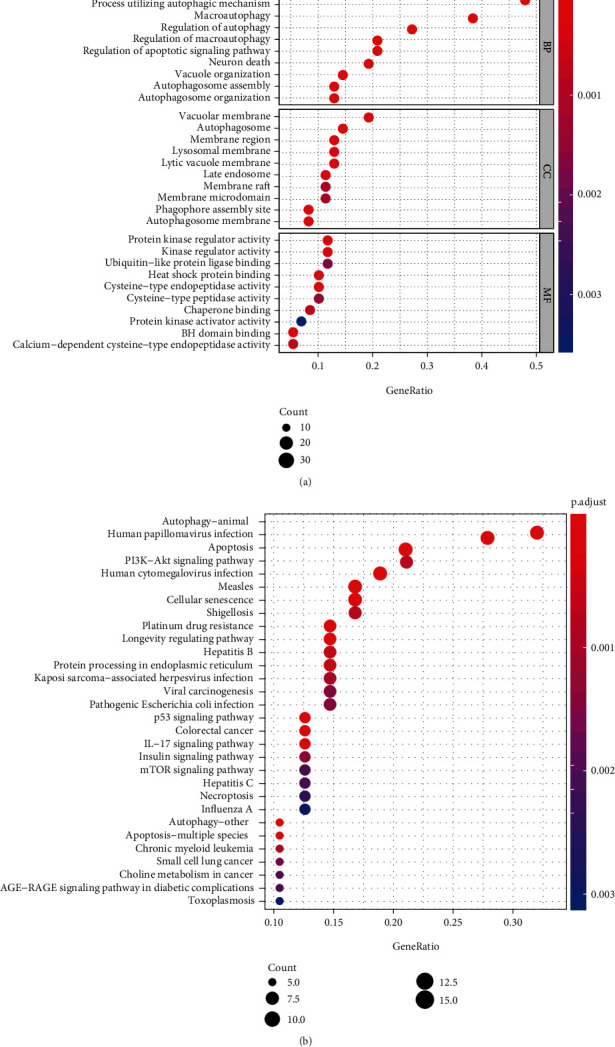
Gene enrichment analysis of GO (a) and KEGG (b). Notes: GO: Gene Ontology; KEGG: Kyoto Encyclopedia of Genes and Genomes.

Gene enrichment analysis can analyze the corresponding biological functions and pathways of selected genes. The biological process of GO is mainly enriched in autophagy, process utilizing autophagic mechanism, macroautophagy, regulation of autophagy, regulation of macroautophagy, regulation of apoptotic signaling pathway, autophagosome assembly, autophagosome organization, neuron death, and vacuumed organization (top 10). The cellular components of GO are mainly enriched in autophagosome, vacuolar membrane, phagophore assembly site, autophagosome membrane, membrane region, late endosome, lysosomal membrane, lytic vacuole membrane, membrane raft, and membrane microdomain (top 10). The molecular function of GO is mainly enriched in protein kinase regulator activity, heat shock protein binding, cysteine-type endopeptidase activity, BH domain binding, kinase regulator activity, chaperone binding, calcium-dependent cysteine-type endopeptidase activity, cysteine-type peptidase activity, ubiquitin-like protein ligase binding, and protein kinase activator activity (top 10) (Figure 2(a)). Similarly, the top 10 significantly enriched pathways are autophagy-animal, human papillomavirus infection, apoptosis, platinum drug resistance, longevity regulated pathway, autophagy-other, apoptosis-multiple species, measles, p53 signaling pathway, and cellular senescence (Figure 2(b)).

### 3.4. Identification of Autophagy-Related Risk Genes for Overall Survival

**Figure 3:**
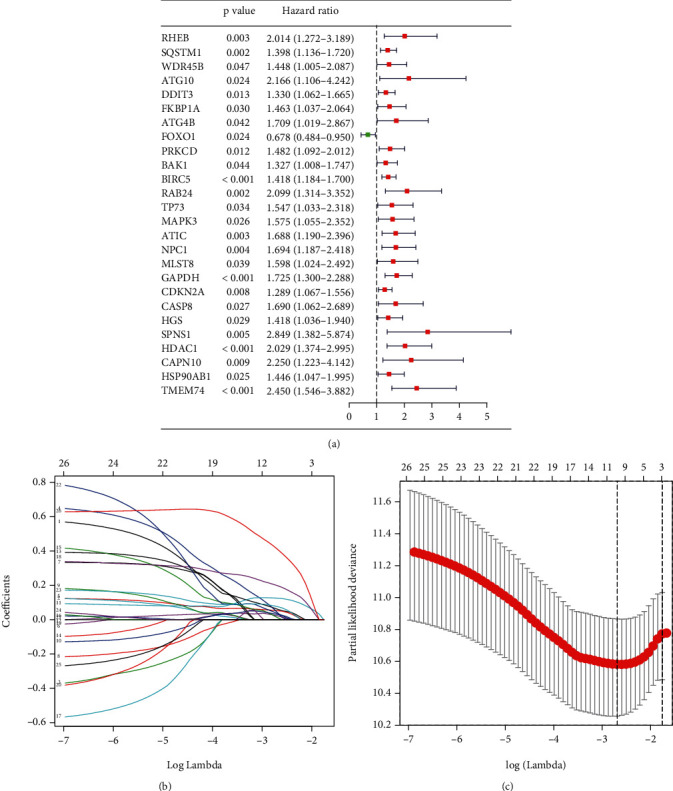
Univariate and LASSO regression analysis. (a) Univariate regression analysis of autophagy-related genes that can significantly affect OS in the TCGA training group. Notes: OS: overall survival; HCC: hepatocellular carcinoma. (b, c) LASSO regression analysis of autophagy-related genes that can significantly affect OS in the TCGA training group. Notes: LASSO: least absolute shrinkage and selection operator; OS: overall survival; TCGA: The Cancer Genome Atlas.

We performed univariate Cox regression analysis on 62 autophagy-related genes in the TCGA training group. The results of the univariate regression analysis showed that 26 autophagy-related genes were significantly associated with the prognosis of HCC patients (*P* < 0.05) (Figure 3(a)). In order to eliminate the false-positive results, we used the LASSO regression model to screen the independent variables further. The results of the LASSO regression analysis showed that 11 genes were significantly correlated with OS (Figures 3(b) and 3(c)). Finally, we put the selected 11 genes into multivariate regression analysis using the backward and forward method. The results of multivariate regression analysis identified ATG10, BIRC5, GAPDH, and TMEM74 are risk factors for OS in the TCGA training group (Table 2).

### 3.5. Development of Autophagy-Related Risk Gene Signature for Overall Survival

**Figure 4:**
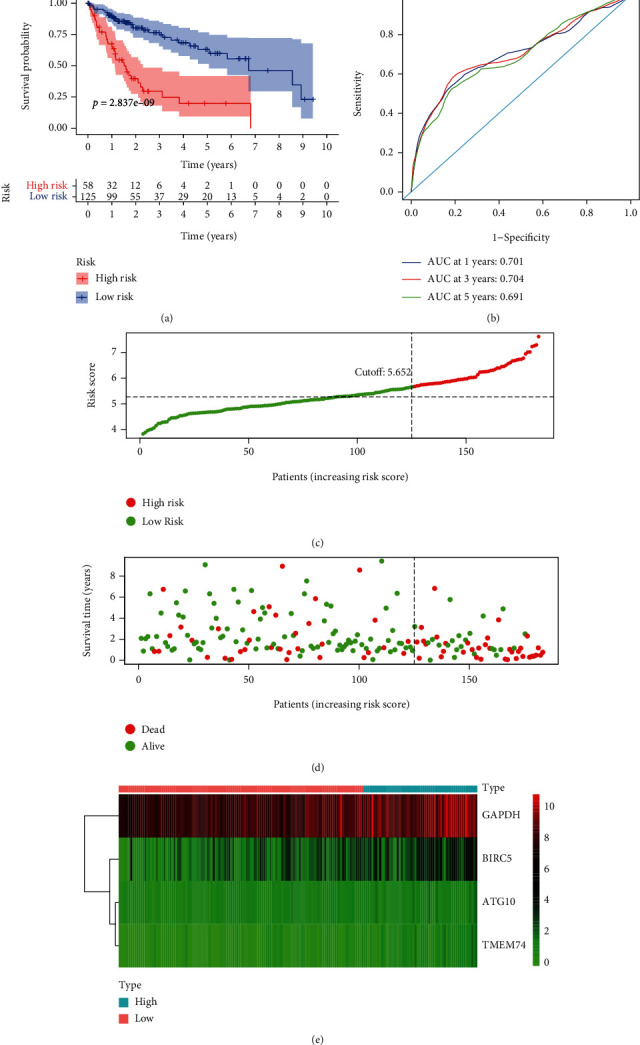
Autophagy-related gene signature for OS in the TCGA training group. (a) Prognostic value of the autophagy-related gene signature for OS in the TCGA training group; (b) diagnostic performance of the autophagy-related gene signature for OS in the TCGA training group; (c) the rank of the risk scores for HCC patients in the TCGA training group; (d) the survival status and time of HCC patients between the high- and low-risk groups for OS in the TCGA training group; (e) the heat map of the autophagy-related risk gene expression matrix for HCC patients in the high- and low-risk groups in the TCGA training group. Notes: OS: overall survival; HCC: hepatocellular carcinoma; TCGA: The Cancer Genome Atlas.

Based on the previous results of the multivariate regression analysis, we constructed a risk score formula for ARGs, which was calculated as follows: risk score = 0.556∗ATG10 + 0.217∗BIRC5 + 0.403∗GAPDH + 0.765∗TMEM74 for OS. According to the regression coefficient, it is evident that ATG10, BIRC5, GAPDH, and TMEM74 are all risk factors for HCC patients. According to the optimal cutoff value (5.652) of a risk score for the autophagy-related gene signature, we divided the TCGA HCC training cohort into high-risk and low-risk groups. The 1-year OS, 3-year OS, and 5-year OS rates of the high-risk group were remarkably lower than the 1-year OS, 3-year OS, and 5-year OS of the low-risk group (63.4% vs. 90.1%; 29.7% vs. 74.5%; 19.8% vs. 62.8%) (*P* = 2.837*e* − 09) (Figure 4(a)). The 1-year AUC, 3-year AUC, and 5-year AUC of the autophagy-related gene signature for OS are 0.701, 0.704, and 0.691 in the TCGA training group (Figure 4(b)). Furthermore, we ranked the prognostic risk scores for OS in the TCGA training group. (Figure 4(c)).  Figure 4(d) shows the survival status and time of HCC patients for OS in the TCGA training group.  Figure 4(e) shows the heat map of the risk ARG expression of HCC patients in the TCGA training group.

### 3.6. Validation of Autophagy-Related Risk Gene Signature for Overall Survival

**Figure 5:**
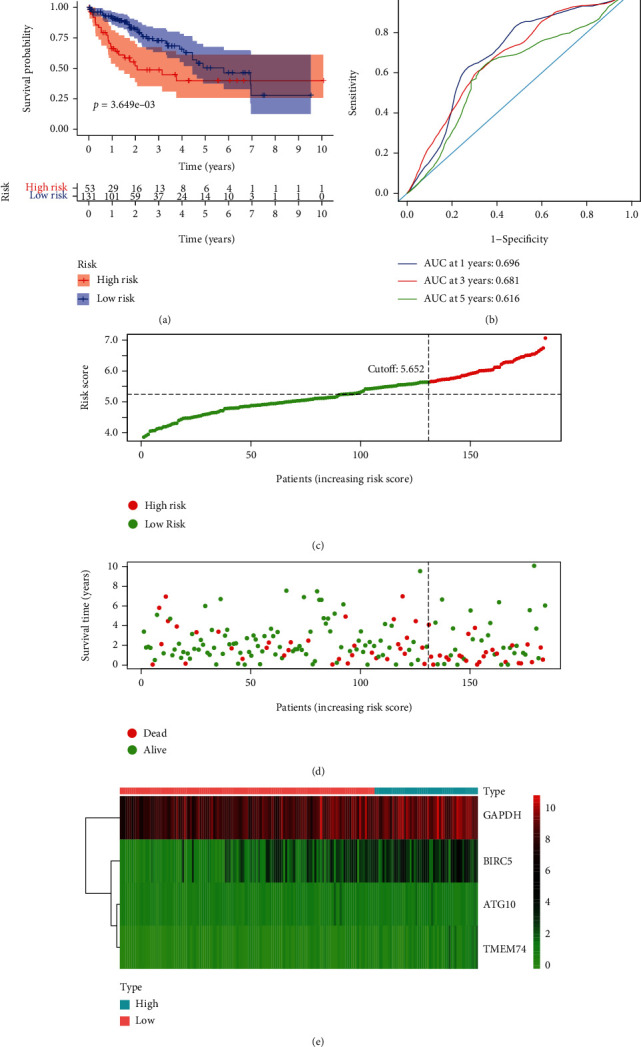
Autophagy-related gene signature for OS in the TCGA testing group. (a) Prognostic value of the autophagy-related gene signature for OS in the TCGA testing group; (b) diagnostic performance of the autophagy-related gene signature for OS in the TCGA testing group; (c) the rank of the risk scores for HCC patients in the TCGA testing group; (d) the survival status and time of HCC patients between the high- and low-risk groups for OS in the TCGA testing group; (e) the heat map of the autophagy-related risk gene expression matrix for HCC patients in the high- and low-risk groups in the TCGA testing group. Notes: OS: overall survival; HCC: hepatocellular carcinoma; TCGA: The Cancer Genome Atlas.

**Figure 6:**
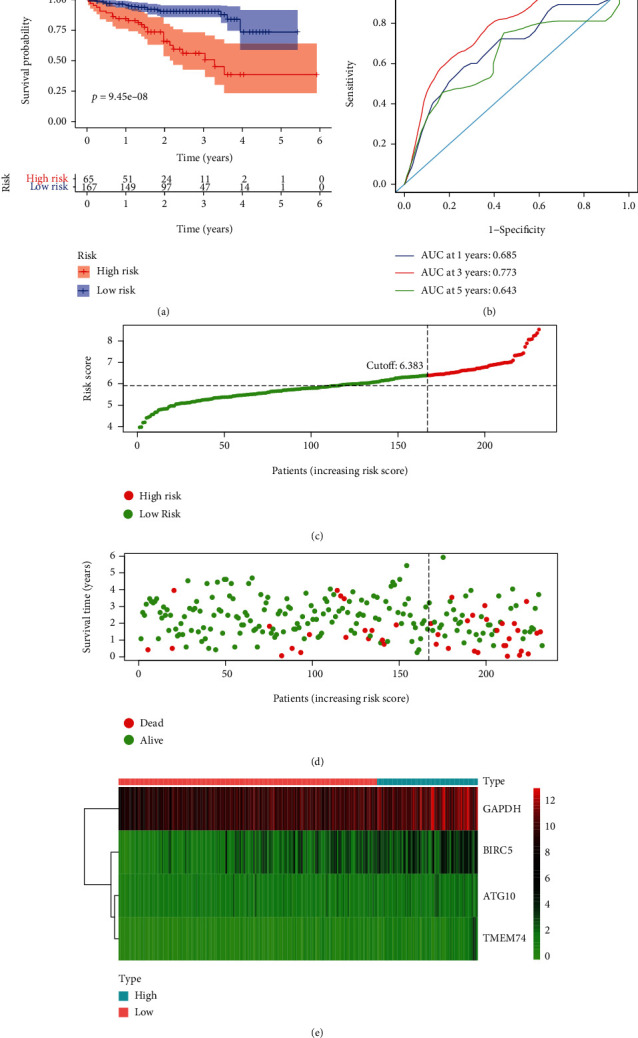
Autophagy-related gene signature for OS in the ICGC HCC dataset. (a) Prognostic value of the autophagy-related gene signature for OS in the ICGC HCC dataset; (b) diagnostic performance of the autophagy-related gene signature for OS in the ICGC HCC dataset; (c) the rank of the risk scores for HCC patients in the ICGC HCC dataset; (d) the survival status and time of HCC patients between the high- and low-risk groups for OS in the ICGC HCC dataset; (e) the heat map of the autophagy-related risk gene expression matrix for HCC patients in the high- and low-risk groups in the ICGC HCC dataset. Notes: OS: overall survival; HCC: hepatocellular carcinoma; ICGC: International Cancer Genome Consortium.

**Figure 7:**
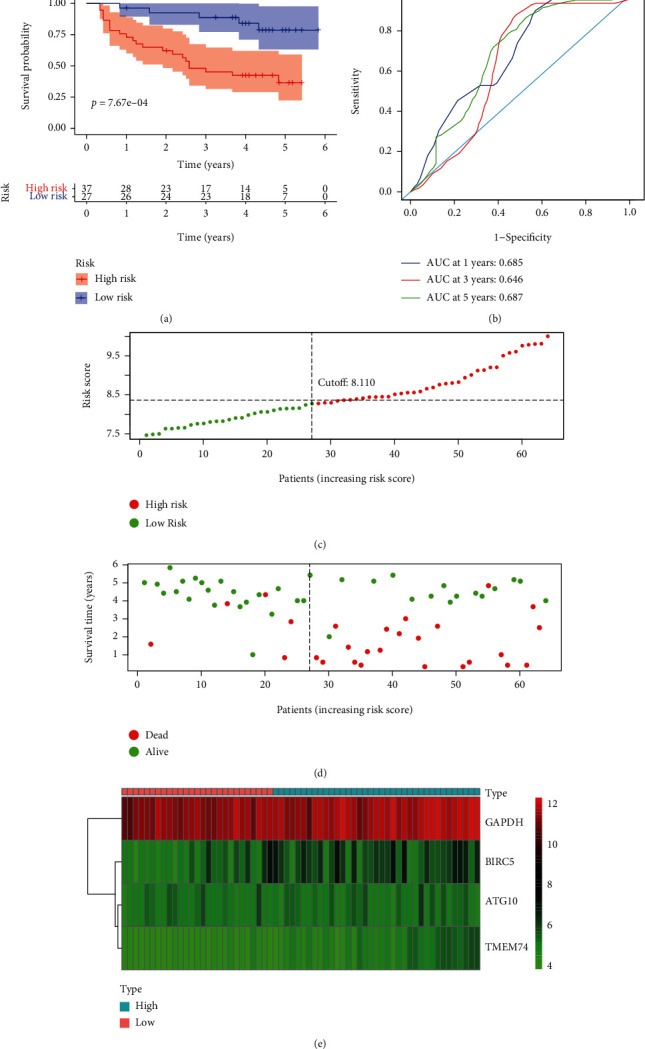
Autophagy-related gene signature for OS in the GSE116174 HCC dataset. (a) Prognostic value of the autophagy-related gene signature for OS in the GSE116174 HCC dataset; (b) diagnostic performance of the autophagy-related gene signature for OS in the GSE116174 HCC dataset; (c) the rank of the risk scores for HCC patients in the GSE116174 HCC dataset; (d) the survival status and time of HCC patients between the high- and low-risk groups for OS in the GSE116174 HCC dataset; (e) the heat map of the autophagy-related risk gene expression matrix for HCC patients in the high- and low-risk groups in the GSE116174 HCC dataset. Notes: OS: overall survival; HCC: hepatocellular carcinoma.

**Figure 8:**
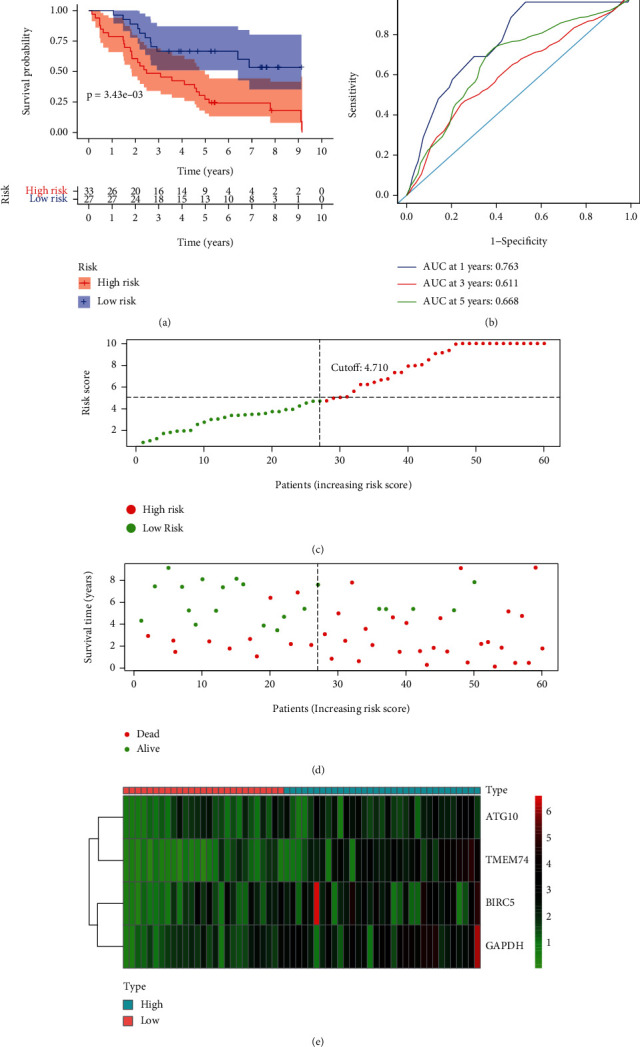
Autophagy-related gene signature for OS in the AHMU HCC dataset. (a) Prognostic value of the autophagy-related gene signature for OS in the AHMU HCC dataset; (b) diagnostic performance of the autophagy-related gene signature for OS in the AHMU HCC dataset; (c) the rank of the risk scores for HCC patients in the AHMU HCC dataset; (d) the survival status and time of HCC patients between the high- and low-risk groups for OS in the AHMU HCC dataset; (e) the heat map of the autophagy-related risk gene expression matrix for HCC patients in the high- and low-risk groups in the AHMU HCC dataset. Notes: OS: overall survival; AHMU: Anhui Medical University; HCC: hepatocellular carcinoma.

The same cutoff value (5.652) of risk score in the TCGA training group was applied to the TCGA testing group. In the TCGA testing group, the 1-year OS, 3-year OS, and 5-year OS rates of the high-risk group were remarkably lower than the 1-year OS, 3-year OS, and 5-year OS of the low-risk group (66.1% vs. 90.9%; 48.8% vs. 72.5%; 39.7% vs. 50.4%) (*P* = 3.649*e* − 3) (Figure 5(a)). The 1-year AUC, 3-year AUC, and 5-year AUC of the autophagy-related gene signature for OS are 0.696, 0.681, and 0.616 (Figure 5(b)). The risk score, survival status, and survival time of HCC patients in the TCGA testing group are plotted in Figures 5(c) and 5(d).  Figure 5(e) shows the heat map of the ARG expression matrix of the HCC patients in the TCGA testing group. To further validate the role of our established formula, we explored the prognostic significance of autophagy-related gene signature in the external ICGC dataset, GSE116174 dataset, and AHMU dataset. In the ICGC dataset, the overall survival rate of the high-risk group was remarkably lower than that of the low-risk group (*P* = 9.45*e* − 8) (Figure 6(a)). The 1-year AUC, 3-year AUC, and 5-year AUC of the autophagy-related gene signature for OS are 0.685, 0.773, and 0.643 (Figure 6(b)). The risk score, survival status, survival time of HCC patients in the ICGC dataset are plotted in Figures 6(c) and 6(d).  Figure 6(e) shows the heat map of the ARG expression matrix of the HCC patients in the ICGC dataset. The same tendency can be found in the GSE116174 dataset. The overall survival rate of the high-risk group was remarkably lower than that of the low-risk group (*P* = 7.67*e* − 4) (Figure 7(a)). The 1-year AUC, 3-year AUC, and 5-year AUC of the autophagy-related gene signature for OS is 0.685, 0.646, and 0.687 (Figure 7(b)). The risk score, survival status, and survival time of HCC patients in the GSE116174 dataset are plotted in Figures 7(c) and 7(d).  Figure 7(e) shows the heat map of the ARG expression matrix of the HCC patients in the GSE116174 dataset. Finally, we used our own dataset for verification. In the AHMU dataset, when compared with the low-risk group, patients in the high-risk group have a poorer prognosis (*P* = 3.43*e* − 3) (Figure 8(a)), and the autophagy-related gene signature shows a better predictive value for HCC (Figure 8(b)). The risk score, survival status, and survival time of HCC patients in the AHMU dataset are plotted in Figures 8(c) and 8(d).  Figure 8(e) shows the heat map of the ARG expression matrix of the HCC patients in the AHMU dataset.

### 3.7. Autophagy-Correlated Gene Signature Is Independently Correlated with Overall Survival

To explore whether the ARG risk score is an independent risk factor for the prognosis of HCC patients in the TCGA training group, we included age, gender, AJCC stage, pathological grade, and the ARG risk score into the univariate regression model. Univariate regression analysis showed that the AJCC stage (*P* = 0.007) and ARG risk score (*P* < 0.001) were significantly associated with OS. Then, we included these factors into multivariate regression analysis. The results of the multivariate regression analysis showed that the AJCC stage (*P* = 0.042) and ARG risk score (*P* < 0.001) were significantly associated with OS (Table 3). Similarly, the AJCC stage and autophagy risk score were independent risk factors for the prognosis of HCC patients in the TCGA testing dataset (Table 4). Besides, in the external validation dataset, the autophagy risk score was also an independent risk factor for the prognosis of HCC patients in the ICGC dataset, GSE116174 dataset, and AHMU dataset (Tables 5–7). Therefore, the ARG risk score is an independent prognostic factor for OS in HCC patients.

### 3.8. Comparison of the Gene Signature We Constructed with the Published Gene Signatures

We also used the TCGA, ICGC, and GSE116174 datasets to analyze the predictive value of gene signature in the two published literature [12, 13] and the gene signature we constructed (Supplementary Figure 2). In the TCGA dataset, the results of the time-dependent ROC curve show that the AUC of 8-gene signature (zhu-signature) [12] for 1-year and 5-year AUC is slightly higher than that of 4-gene signature (kong-signature) and 3-gene signature (lin-signature) [13], but its 3-year AUC is slighter lower than kong-signature and lin-signature. The predicted value of 1-year, 3-year, and 5-year AUC is similar between kong-signature and lin-signature. In the ICGC dataset, the 1-year AUC of kong-signature is slightly lower than zhu-signature but was higher than lin-signature and zhu-signature in both 3-year and 5-year AUC. In the GSE116174 dataset, kong-signature has shown good predictive value in 1-year, 3-year, and 5-year AUC. In summary, the kong-signature we constructed has a more accurate predictive value than the two other gene signatures in the previously published literature.

## 4. Discussion

With the development of science and technology in recent years, autophagy has gradually attracted the attention of researchers as an essential molecular process. Although many reports have explored the role of individual autophagy-related genes in liver cancer, research on the prognostic role of all autophagy-related genes in HCC is rarely investigated. Therefore, we deeply explored autophagy-related risk genes that affect the prognosis of HCC patients by digging into multiple public databases, so as to provide guidance for clinical evaluation of patients with hepatocellular carcinoma.

In our study, we downloaded and analyzed the RNA-seq data of the HCC cohort from TCGA and obtained 62 differentially expressed autophagy-related genes using the Wilcoxon test. Then, we performed gene enrichment analysis, and the function and pathway of autophagy-related genes play an essential role in the progression of hepatocellular carcinoma. Furthermore, we used univariate regression analysis to explore the relationship between the expression of autophagy-related genes and the overall survival of HCC patients. The results showed that 26 autophagy-related genes were significantly related to OS. The LASSO regression and multivariate regression analysis were utilized for further screening, and we found that ATG10, BIRC5, GAPDH, and TMEM74 are risk ARGs for OS. Therefore, we established a prognostic gene signature for OS in the TCGA training group. The prognostic role of autophagy-related gene signature was also validated in the TCGA testing group, ICGC dataset, GSE116174 dataset, and AHMU dataset. Finally, we compared the gene signature we built with the published gene signatures. In our study, based on different statistical methods, we constructed a new 4-gene signature for the HCC prediction model. In both the internal training dataset and the external validation dataset, the 4-gene signature has better robustness and accuracy compared to the other two predictive signatures, which provides a new strategy for predicting the prognosis of liver cancer patients.

ATG10, also named ATG10L, encodes a protein that is involved in Ub-like modification, which is crucial for the formation of autophagosomes. ATG10 has been explored in a variety of tumors, including colorectal cancer, gastric cancer, non-small-cell lung cancer, and breast cancer [14–17]. For example, Jo et al.'s study found that increased expression of ATG10 is associated with vascular invasion, lymphatic metastasis, and poor prognosis in colorectal cancer [15]. Xie et al.'s research found that ATG10 can promote the proliferation and migration of lung cancer [17]. This is consistent with our study's finding that elevated expression of ATG10 is consistent with poor prognosis in patients with HCC.

BIRC5, also known as survivin, is a critical member of the apoptosis-inhibiting gene family, and it can encode regulatory molecules that inhibit the death of apoptosis cells. The abnormal expression of BIRC5 is related to the malignant transformation of the cancer cell [18–23]. For example, the high expression of BIRC5 can promote the proliferation and angiogenesis of liver cancer cells, reduce the sensitivity to chemotherapy and radiotherapy, and suppress the apoptosis of tumor cells, thereby affecting the survival outcome of HCC patients [18]. Our study found that the high expression of BIRC5 is associated with poor prognosis in patients with HCC, which is also consistent with the published literature.

GAPDH is a well-known housekeeping gene. It includes a C-terminal catalytic domain and an N-terminal NAD + binding domain and plays an important role in the body's energy metabolism, DNA repair, autophagy, and cell proliferation [24–26]. Liu et al.'s research finds that GAPDH can convert glycolytic flux to serine metabolism by increasing PHGDH and promoting histone methylation, thereby promoting the progress of liver tumors in mice [26]. Ganapathy-Kanniappan et al.'s research found that injection of GAPDH antagonists into mouse liver cancer models can induce apoptosis of liver cancer cells and block the progression of Hep3B tumors, which may be used as a potential target therapy for HCC [25]. In our study, GAPDH was significantly upregulated in liver cancer, and high expression of GAPDH was an independent risk factor for prognosis in patients with liver cancer, which is consistent with the literature.

TMEM74, also known as FLJ30668 or NET36, is an autophagosome protein and lysosomal. It contains two TM domains and was first discovered by Yu et al. [27]. It plays a role in apoptosis, autophagy, tumor progression, and neurological diseases [28–32]. Sun et al.'s research finds that TMEM74 can induce autophagy through interaction with ATG16L1 and ATG9A, thereby promoting tumor cell survival [30]. Sun et al.'s study found that TMEM74 is highly expressed in liver cancer and lung cancer tissues and correlated to the poor prognosis of cancer patients. After overexpressing TMEM74 in tumor cells, the proliferation ability of tumor cells was enhanced [31]. In our study, elevated expression of TEME74 was associated with poor prognosis in patients with liver cancer, which is in accordance with the study by Sun et al.

However, there are still deficiencies in our research. Firstly, the data we use is derived from public databases, and these findings require subsequent verification. Secondly, we have discovered some potential biomarkers of liver cancer and have not further explored the underlying mechanism of its function, and subsequent research is needed to explore it.

## 5. Conclusion

In this research, we found autophagy-related risk scores can significantly distinguish high- and low-risk groups of HCC patients and are also significantly related to the prognosis of HCC patients. Therefore, the prediction model based on autophagy-related genes may be used to predict the prognosis of HCC patients to provide a new strategy for the prevention of HCC in the clinic.

## Figures and Tables

**Table 1:** Clinicopathological characteristics of the TCGA training group, the TCGA testing group, and the entire TCGA dataset.

Variables	Entire dataset	Training group	Testing group	*P* value
	*n* = 367	*n* = 183	*n* = 184	
Age (years)	61.0 (52.0-69.0)	61.0 (52.0-68.0)	62.0 (51.0-69.8)	0.487
Gender, male/female	248/119	125/58	123/61	0.765
AJCC stage, I/II/III/IV/NA	171/85/83/4/24	86/51/37/1/8	85/34/46/3/16	0.090
Histologic grade, G4/G3/G2/G1/NA	55/176/119/12/5	25/89/61/5/3	30/87/58/7/2	0.897
Survival status, alive/dead	237/130	113/70	124/60	0.258
Survival time (months)	19.7 (11.3-36.3)	18.9 (11.4-34.5)	20.8 (10.6-40.0)	0.758

Notes: AJCC: American Joint Committee on Cancer; TCGA: The Cancer Genome Atlas.

**Table 2:** Multivariate Cox regression analysis of autophagy-related genes that can significantly affect OS in the TCGA training group.

Id	Coef	HR	HR.95L	HR.95H	*P* value
ATG10	0.556	1.744	0.913	3.332	0.092
BIRC5	0.217	1.242	1.019	1.514	0.032
GAPDH	0.403	1.496	1.096	2.040	0.011
TMEM74	0.765	2.150	1.378	3.353	0.001

Notes: HR: hazards ratio; OS: overall survival; HCC: hepatocellular carcinoma.

**Table 3:** Univariate and multivariate regression analyses of OS in HCC patients of the TCGA training group.

Variable	Univariate analysis			Multivariate analysis		
	*P*	Hazard ratio	95% confidence interval	*P*	Hazard ratio	95% confidence interval
Age (≥60/<60)	0.220	1.378	0.826-2.301			
Sex (female/male)	0.776	1.079	0.639-1.822			
AJCC stage (IV + III/II + I)	0.007	2.061	1.216-3.494	0.042	1.745	1.021-2.983
Histologic grade (G4 + G3/G2 + G1)	0.276	0.744	0.437-1.266			
Risk score (high/low)	<0.001	4.027	2.410-6.728	<0.001	3.775	2.251-6.328

Notes: AJCC: American Joint Committee on Cancer; OS: overall survival; HCC: hepatocellular carcinoma; TCGA: The Cancer Genome Atlas.

**Table 4:** Univariate and multivariate regression analyses of OS in HCC patients of the TCGA testing group.

Variable	Univariate analysis			Multivariate analysis		
	*P*	Hazard ratio	95% confidence interval	*P*	Hazard ratio	95% confidence interval
Age (≥60/<60)	0.808	1.071	0.616-1.863			
Sex (female/male)	0.082	1.637	0.940-2.852			
AJCC stage (IV + III/II + I)	<0.001	3.495	2.002-6.100	<0.001	3.545	2.017-6.231
Histologic grade (G4 + G3/G2 + G1)	0.025	1.886	1.084-3.280	0.036	1.831	1.041-3.221
Risk score (high/low)	0.006	2.201	1.259-3.850	0.020	1.947	1.109-3.416

Notes: AJCC: American Joint Committee on Cancer; OS: overall survival; HCC: hepatocellular carcinoma; TCGA: The Cancer Genome Atlas.

**Table 5:** Univariate and multivariate regression analyses of OS in HCC patients of the ICGC dataset.

Variable	Univariate analysis			Multivariate analysis		
	*P* value	Hazard ratio	95% confidence interval	*P* value	Hazard ratio	95% confidence interval
Age (≥60/<60)	0.590	0.823	0.405-1.671			
Sex (female/male)	0.039	1.926	1.033-3.590	0.017	2.231	1.152-4.322
AJCC stage (IV + III/II + I)	0.005	2.384	1.304-4.359	0.002	2.726	1.437-5.173
Prior malignancy (yes/no)	0.180	1.750	0.773-3.963			
Risk (high/low)	<0.001	4.528	2.462-8.328	<0.001	4.084	2.214-7.533

Notes: AJCC: American Joint Committee on Cancer; OS: overall survival; HCC: hepatocellular carcinoma; ICGC: International Cancer Genome Consortium.

**Table 6:** Univariate and multivariate regression analyses of OS in HCC patients of the GSE116174 dataset.

Variable	Univariate analysis			Multivariate analysis		
	*P* value	Hazard ratio	95% confidence interval	*P* value	Hazard ratio	95% confidence interval
Age (≥60/<60)	0.788	0.892	0.387-2.055			
Sex (female/male)	0.250	0.309	0.042-2.287			
Alcohol (yes/no)	0.968	0.980	0.369-2.603			
Smoke (yes/no)	0.339	1.459	0.673-3.163			
AJCC stage (IV + III/II + I)	0.300	1.622	0.651-4.043			
Risk (high/low)	0.003	4.429	1.664-11.788	0.003	4.429	1.664-11.788

Notes: AJCC: American Joint Committee on Cancer; OS: overall survival; HCC: hepatocellular carcinoma.

**Table 7:** Univariate and multivariate regression analyses of OS in HCC patients of the AHMU dataset.

Variable	Univariate analysis			Multivariate analysis		
	*P* value	Hazard ratio	95% confidence interval	*P* value	Hazard ratio	95% confidence interval
Age (≥60/<60)	0.908	0.962	0.497-1.861			
Sex (female/male)	0.664	0.841	0.385-1.839			
AJCC stage (IV + III/II + I)	0.014	2.303	1.184-4.480	**—**		
Cirrhosis (yes/no)	0.330	0.721	0.374-1.391			
Tumor size (>5/≤5 cm)	0.432	1.296	0.679-2.473			
Tumor number (multiple/single)	0.004	2.687	1.365-5.291	0.013	2.372	1.203-4.676
Differentiation (poor/well + moderate)	0.471	1.265	0.667-2.398			
Risk (high/low)	0.005	2.752	1.358-5.575	0.011	2.517	1.238-5.119

Notes: AJCC: American Joint Committee on Cancer; OS: overall survival; HCC: hepatocellular carcinoma.

## Data Availability

We use public databases for subsequent analysis, and the corresponding data can be found in The Human Autophagy Database (HADb), The Cancer Genome Atlas (TCGA), International Cancer Genome Consortium (ICGC), Gene Expression Omnibus (GEO), and the cBioPortal.

## Supplementary Materials

Supplementary Materials Supplementary Figure 1: cluster heat map of differentially expressed autophagy-related genes in HCC patients from the TCGA cohort. Supplementary Figure 2: comparison of the gene signature we constructed with the published gene signatures. Supplementary Table 1: clinicopathological characteristics of the ICGC, GSE116174, and the AHMU dataset. Supplementary Table 2: primers used for PCR.
