# Supplementary material for: Development and Validation of an Autophagy-Related Gene Signature for Predicting the Prognosis of Hepatocellular Carcinoma
Source: Biomed Res Int. 2021 Oct 28;2021:7771037. doi: 10.1155/2021/7771037 (PMC8568514; doi:10.1155/2021/7771037)
Supplement: Supplementary Materials — Supplementary Figure 1: cluster heat map of differentially expressed autophagy-related genes in HCC patients from the TCGA cohort. Supplementary Figure 2: comparison of the gene signature we constructed with the published gene signatures. Supplementary Table 1: clinicopathological characteristics of the ICGC, GSE116174, and the AHMU dataset. Supplementary Table 2: primers used for PCR. [file 7771037.f1.docx]

**Supplementary Figure1**. Cluster heatmap of differentially expressed autophagy-related genes in HCC patients from the TCGA cohort.


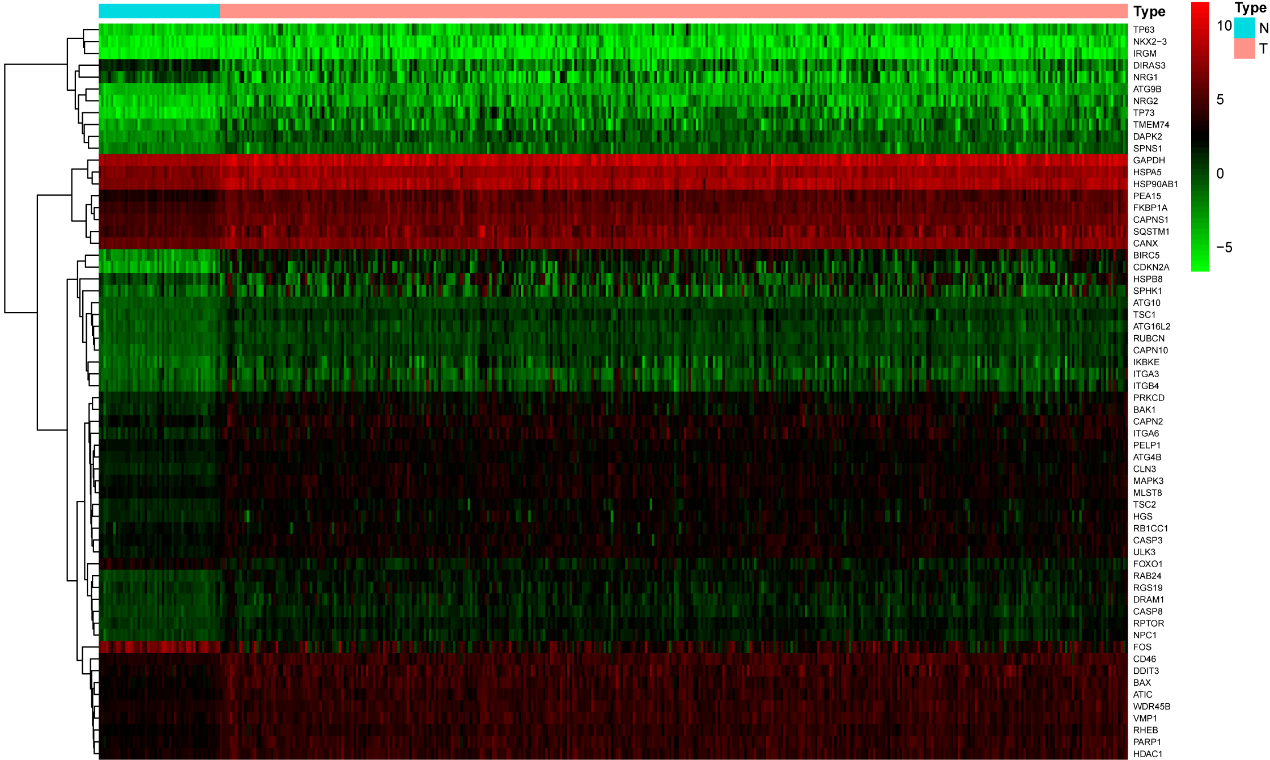
Notes: Heat map of the expression matrix of differentially autophagy-related genes; The horizontal axis represents the samples. The vertical axis represents the differentially expressed autophagy-related genes; TCGA: The Cancer Genome Atlas.

**Supplementary Figure2**. Comparison of the gene signature we constructed with the published gene signatures.


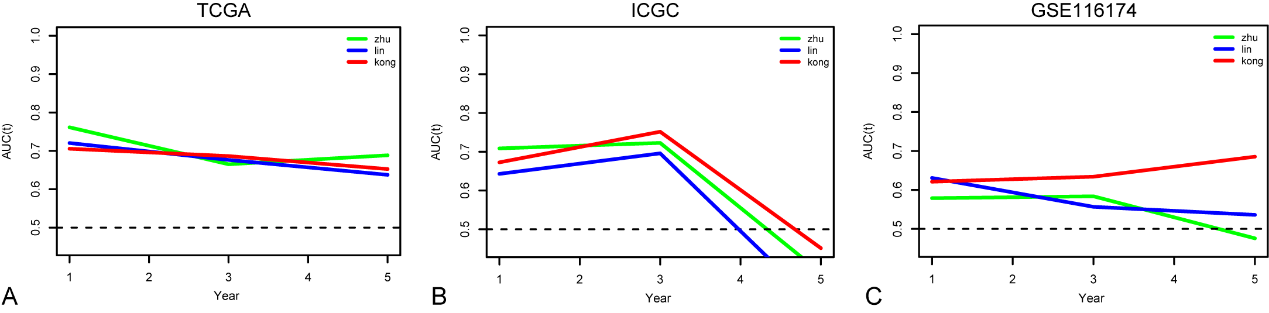


Notes: Kong is the gene signature we constructed, zhu and lin are the gene signature that published in the previous literature；TCGA: The Cancer Genome Atlas; ICGC: International Cancer Genome Consortium.

**Supplementary Table1**. Clinicopathological characteristics of the ICGC, GSE116174, and the AHMU dataset.

| Variables | ICGC | GSE116174 | AHMU |
| --- | --- | --- | --- |
|  | n=232 | n=64 | n=60 |
| Age (years) | 68.5 (62.0-74.0) | 53.5 (49.0-62.0) | 53.3±13.2 |
| Gender, male/female | 171/61 | 58/6 | 47/13 |
| AJCC Stage, I/II/III/IV | 36/106/71/19 | 8/45/11/0 | 29/13/10/8 |
| Histologic grade, well/moderate/poor | - | - | 7/25/28 |
| Prior-maligancy, no/yes | 202/30 | - | - |
| Cirrhosis, no/yes | - | - | 20/40 |
| Tumor size, ≤5/>5 cm | - | - | 32/28 |
| Tumor number, single /multiple | - | - | 46/14 |
| Survival status, Alive/Dead | 189/43 | 37/27 | 21/39 |
| Survival time (months) | 25.6 (16.8-36.2) | 47.5 (21.0-56.0) | 47.0 (22.1-64.8) |

Notes: ICGC: International Cancer Genome Consortium; AHMU: anhui medical university.

| **Supplementary Table 2.** Primers used for PCR | |
| --- | --- |
| Gene | Forward and Reverse primer |
| ATG10 | F: 5' CCAAGGTGGGAGCATCACTT 3' |
|  | R: 5' CACAGTCTCAGCTCACTGCA 3' |
| BIRC5 | F: 5' GGAGTCTGGGAAGGGTTGTG 3' |
|  | R: 5' TGGCTTGCTGGTCTCTTCTG 3' |
| GAPDH | F: 5' ACTCCTCCACCTTTGACGC 3' |
|  | R: 5' GCTGTAGCCAAATTCGTTGTC 3' |
| TMEM74 | F: 5' CTTGAGAGTCTGCCTTGGCA 3' |
|  | R: 5' TGTGGCTGGTTGTGGTTTCT 3' |
| U6 | F: 5' GCTTCGGCAGCACATATACTAAAAT 3' |
|  | R: 5' CGCTTCACGAATTTGCGTGTCAT 3' |

**Supplementary R transcript for this research**

**(1) normality test**

library(data.table)

library(dplyr)

library(tibble)

options(digits = 3)

dd <- fread('ARGexp.txt',data.table = F)%>%

column_to_rownames('id')%>%

t()%>%

as.data.frame()

result <- data.frame()

for (i in 1:ncol(dd)) {

fit <- ks.test(as.numeric(dd[,i]),"pnorm")

p1 <- fit$p.value

fit2 <- ks.test(log2(as.numeric(dd[,i])+1),"pnorm")

p2 <- fit2$p.value

result <- rbind(result,data.frame(ID=colnames(dd)[i],nonlog_P=p1,log_P=p2))

}

save(result,file = 'result.rda')

**(2)** **differentially expressed genes**

library("limma")

setwd("C:\\Users\\Administrator\\Desktop\\hcczisi\\08.diff")

inputFile="ARGexp.txt"

fdrFilter=0.05

logFCfilter=1

conNum=50

treatNum=374

outTab=data.frame()

grade=c(rep(1,conNum),rep(2,treatNum))

rt=read.table(inputFile,sep="\t",header=T,check.names=F)

rt=as.matrix(rt)

rownames(rt)=rt[,1]

exp=rt[,2:ncol(rt)]

dimnames=list(rownames(exp),colnames(exp))

data=matrix(as.numeric(as.matrix(exp)),nrow=nrow(exp),dimnames=dimnames)

data=avereps(data)

data=data[rowMeans(data)>0,]

for(i in row.names(data)){

geneName=unlist(strsplit(i,"\\|",))[1]

geneName=gsub("\\/", "_", geneName)

rt=rbind(expression=data[i,],grade=grade)

rt=as.matrix(t(rt))

wilcoxTest<-wilcox.test(expression ~ grade, data=rt)

conGeneMeans=mean(data[i,1:conNum])

treatGeneMeans=mean(data[i,(conNum+1):ncol(data)])

logFC=log2(treatGeneMeans)-log2(conGeneMeans)

pvalue=wilcoxTest$p.value

conMed=median(data[i,1:conNum])

treatMed=median(data[i,(conNum+1):ncol(data)])

diffMed=treatMed-conMed

if( ((logFC>0) & (diffMed>0)) | ((logFC<0) & (diffMed<0)) ){

outTab=rbind(outTab,cbind(gene=i,conMean=conGeneMeans,treatMean=treatGeneMeans,logFC=logFC,pValue=pvalue))

}

}

pValue=outTab[,"pValue"]

fdr=p.adjust(as.numeric(as.vector(pValue)),method="fdr")

outTab=cbind(outTab,fdr=fdr)

write.table(outTab,file="all.xls",sep="\t",row.names=F,quote=F)

outDiff=outTab[( abs(as.numeric(as.vector(outTab$logFC)))>logFCfilter & as.numeric(as.vector(outTab$fdr))<fdrFilter),]

write.table(outDiff,file="diff.xls",sep="\t",row.names=F,quote=F)

heatmap=rbind(ID=colnames(data[as.vector(outDiff[,1]),]),data[as.vector(outDiff[,1]),])

write.table(heatmap,file="diffAutophagyExp.txt",sep="\t",col.names=F,quote=F)

pdf(file="vol.pdf",height=5,width=5)

xMax=max(abs(as.numeric(as.vector(outTab$logFC))))

yMax=max(-log10(outTab$fdr))+1

plot(as.numeric(as.vector(outTab$logFC)), -log10(outTab$fdr), xlab="logFC",ylab="-log10(fdr)",

main="Volcano", ylim=c(0,yMax),xlim=c(-xMax,xMax),yaxs="i",pch=20, cex=0.8)

diffSub=subset(outTab, fdr<fdrFilter & as.numeric(as.vector(logFC))>logFCfilter)

points(as.numeric(as.vector(diffSub$logFC)), -log10(diffSub$fdr), pch=20, col="red",cex=0.8)

diffSub=subset(outTab, fdr<fdrFilter & as.numeric(as.vector(logFC))<(-logFCfilter))

points(as.numeric(as.vector(diffSub$logFC)), -log10(diffSub$fdr), pch=20, col="green",cex=0.8)

abline(v=0,lty=2,lwd=3)

dev.off()

library(pheatmap)

hmExp=data[as.vector(outDiff[,1]),]

hmExp=log2(hmExp+0.01)

Type=c(rep("N",conNum),rep("T",treatNum))

names(Type)=colnames(data)

Type=as.data.frame(Type)

pdf(file="heatmap.pdf",height=6,width=10)

pheatmap(hmExp,

annotation=Type,

color = colorRampPalette(c("green", "black", "red"))(50),

cluster_cols =F,

show_colnames = F,

show_rownames = T,

fontsize = 8,

fontsize_row=4.5,

fontsize_col=5)

dev.off()

**(3) GO**

library("clusterProfiler")

library("org.Hs.eg.db")

library("enrichplot")

library("ggplot2")

setwd("C:\\Users\\Administrator\\Desktop\\hcczisi\\11.GO")

rt=read.table("id.txt",sep="\t",header=T,check.names=F)

rt=rt[is.na(rt[,"entrezID"])==F,]

gene=rt$entrezID

kk <- enrichGO(gene = gene,

OrgDb = org.Hs.eg.db,

pvalueCutoff =0.05,

qvalueCutoff = 0.05,#????0.05û?н??????Ͱ?qvalue????Ϊ1??0.05???á?

ont="all", #BP CC MF?????֣????Ե???????

readable =T)

write.table(kk,file="GO.txt",sep="\t",quote=F,row.names = F)

pdf(file="barplot.pdf",width = 10,height = 7)

barplot(kk, drop = TRUE, showCategory =10,split="ONTOLOGY") + facet_grid(ONTOLOGY~., scale='free')

dev.off()

pdf(file="bubble.pdf",width = 10,height = 7)

dotplot(kk,showCategory = 10,split="ONTOLOGY") + facet_grid(ONTOLOGY~., scale='free')

dev.off()

**(4) KEGG**

library("clusterProfiler")

library("org.Hs.eg.db")

library("enrichplot")

library("ggplot2")

setwd("C:\\Users\\Administrator\\Desktop\\hcczisi\\13.KEGG")

rt=read.table("id.txt",sep="\t",header=T,check.names=F)

rt=rt[is.na(rt[,"entrezID"])==F,]

gene=rt$entrezID

kk <- enrichKEGG(gene = gene, organism = "hsa", pvalueCutoff =0.05, qvalueCutoff =1)

write.table(kk,file="KEGGId.txt",sep="\t",quote=F,row.names = F)

pdf(file="barplot.pdf",width = 10,height = 8)

barplot(kk, drop = TRUE, showCategory = 30)

dev.off()

pdf(file="bubble.pdf",width = 10,height = 8)

dotplot(kk, showCategory = 30)

dev.off()

**(5) Univariate Cox regression analysis**

library(survival)

pFilter=0.05

setwd("C:\\Users\\Administrator\\Desktop\\hcczisi\\16.uniCox")

rt=read.table("all.txt",header=T,sep="\t",check.names=F,row.names=1)

rt=read.table("expTime.txt",header=T,sep="\t",check.names=F,row.names=1)

num=nrow(rt)

set.seed(123) #random number generator

ind <- sample(x=1:num, size=num*0.5, replace = FALSE)

train <- rt[ind,] #the training data set

test <- rt[-ind,] #the test data set

write.table(train,file="train.txt",sep="\t",row.names=TRUE,quote=F)

write.table(test,file="test.txt",sep="\t",row.names=TRUE,quote=F)

outTab=data.frame()

sigGenes=c("futime","fustat")

rt[,3:ncol(rt)]=log2(rt[,3:ncol(rt)]+1)

for(i in colnames(rt[,3:ncol(rt)])){

cox <- coxph(Surv(futime, fustat) ~ rt[,i], data = rt)

coxSummary = summary(cox)

coxP=coxSummary$coefficients[,"Pr(>|z|)"]

if(coxP<pFilter){

sigGenes=c(sigGenes,i)

outTab=rbind(outTab,

cbind(id=i,

HR=coxSummary$conf.int[,"exp(coef)"],

HR.95L=coxSummary$conf.int[,"lower .95"],

HR.95H=coxSummary$conf.int[,"upper .95"],

pvalue=coxSummary$coefficients[,"Pr(>|z|)"])

)

}

}

write.table(outTab,file="uniCox.txt",sep="\t",row.names=F,quote=F)

uniSigExp=rt[,sigGenes]

uniSigExp=cbind(id=row.names(uniSigExp),uniSigExp)

write.table(uniSigExp,file="uniSigExp.txt",sep="\t",row.names=F,quote=F)

rt <- read.table("uniCox.txt",header=T,sep="\t",row.names=1,check.names=F)

gene <- rownames(rt)

hr <- sprintf("%.3f",rt$"HR")

hrLow <- sprintf("%.3f",rt$"HR.95L")

hrHigh <- sprintf("%.3f",rt$"HR.95H")

Hazard.ratio <- paste0(hr,"(",hrLow,"-",hrHigh,")")

pVal <- ifelse(rt$pvalue<0.001, "<0.001", sprintf("%.3f", rt$pvalue))

pdf(file="forest.pdf", width = 10,height = 10)

n <- nrow(rt)

nRow <- n+1

ylim <- c(1,nRow)

layout(matrix(c(1,2),nc=2),width=c(3,2))

xlim = c(0,3)

par(mar=c(4,2.5,2,1))

plot(1,xlim=xlim,ylim=ylim,type="n",axes=F,xlab="",ylab="")

text.cex=0.8

text(0,n:1,gene,adj=0,cex=text.cex)

text(1.5-0.5*0.2,n:1,pVal,adj=1,cex=text.cex);text(1.5-0.5*0.2,n+1,'pvalue',cex=text.cex,font=2,adj=1)

text(3,n:1,Hazard.ratio,adj=1,cex=text.cex);text(3,n+1,'Hazard ratio',cex=text.cex,font=2,adj=1,)

\

par(mar=c(4,1,2,1),mgp=c(2,0.5,0))

xlim = c(0,max(as.numeric(hrLow),as.numeric(hrHigh)))

plot(1,xlim=xlim,ylim=ylim,type="n",axes=F,ylab="",xaxs="i",xlab="Hazard ratio")

arrows(as.numeric(hrLow),n:1,as.numeric(hrHigh),n:1,angle=90,code=3,length=0.05,col="darkblue",lwd=2.5)

abline(v=1,col="black",lty=2,lwd=2)

boxcolor = ifelse(as.numeric(hr) > 1, 'red', 'green')

points(as.numeric(hr), n:1, pch = 15, col = boxcolor, cex=1.3)

axis(1)

dev.off()

**(6) Lasso regression**

library("glmnet")

library("survival")

setwd("C:\\Users\\Administrator\\Desktop\\hcczisi\\17.multiCox")

rt=read.table("uniSigExp.txt",header=T,sep="\t",row.names=1)

rt$futime=rt$futime/12

gene=read.table("gene.txt",header=F)

rt=rt[,c("futime","fustat",as.vector(gene[,1]))]

x=as.matrix(rt[,c(3:ncol(rt))])

y=data.matrix(Surv(rt$futime,rt$fustat))

fit <- glmnet(x, y, family = "cox", maxit = 1000)

pdf("lambda.pdf")

plot(fit, xvar = "lambda", label = TRUE)

dev.off()

cvfit <- cv.glmnet(x, y, family="cox", maxit = 1000)

pdf("cvfit.pdf")

plot(cvfit)

abline(v=log(c(cvfit$lambda.min,cvfit$lambda.1se)),lty="dashed")

dev.off()

coef <- coef(fit, s = cvfit$lambda.min)

index <- which(coef != 0)

actCoef <- coef[index]

lassoGene=row.names(coef)[index]

geneCoef=cbind(Gene=lassoGene,Coef=actCoef)

write.table(geneCoef,file="geneCoef.txt",sep="\t",quote=F,row.names=F)

riskScore=predict(cvfit, newx = x, s = "lambda.min",type="response")

outCol=c("futime","fustat",lassoGene)

risk=as.vector(ifelse(riskScore>median(riskScore),"high","low"))

outTab=cbind(rt[,outCol],riskScore=as.vector(riskScore),risk)

write.table(cbind(id=rownames(outTab),outTab),

file="lassoRisk.txt",

sep="\t",

quote=F,

row.names=F)

**(7) Multivariate Cox regression analysis**

library(survival)

setwd("C:\\Users\\Administrator\\Desktop\\hcczi\\17.multiCox")

rt=read.table("lassoRisk.txt",header=T,sep="\t",check.names=F,row.names=1)

#rt$futime=rt$futime/12

multiCox=coxph(Surv(futime, fustat) ~ ., data = rt)

multiCox=step(multiCox,direction = "both")

multiCoxSum=summary(multiCox)

outTab=data.frame()

outTab=cbind(

coef=multiCoxSum$coefficients[,"coef"],

HR=multiCoxSum$conf.int[,"exp(coef)"],

HR.95L=multiCoxSum$conf.int[,"lower .95"],

HR.95H=multiCoxSum$conf.int[,"upper .95"],

pvalue=multiCoxSum$coefficients[,"Pr(>|z|)"])

outTab=cbind(id=row.names(outTab),outTab)

outTab=gsub("`","",outTab)

write.table(outTab,file="multiCox.xls",sep="\t",row.names=F,quote=F)

riskScore=predict(multiCox,type="risk",newdata=rt)

coxGene=rownames(multiCoxSum$coefficients)

coxGene=gsub("`","",coxGene)

outCol=c("futime","fustat",coxGene)

risk=as.vector(ifelse(riskScore>median(riskScore),"high","low"))

write.table(cbind(id=rownames(cbind(rt[,outCol],riskScore,risk)),cbind(rt[,outCol],riskScore,risk)),

file="risk.txt",

sep="\t",

quote=F,

row.names=F)

**(8) Risk Plot**

library(pheatmap)

setwd("C:\\Users\\Administrator\\Desktop\\autophagy\\19.riskPlot")

rt=read.table("icgcrisk.txt",sep="\t",header=T,row.names=1,check.names=F)

rt=rt[order(rt$riskScore),]

riskClass=rt[,"risk"]

lowLength=length(riskClass[riskClass=="low"])

highLength=length(riskClass[riskClass=="high"])

line=rt[,"riskScore"]

line[line>10]=10

pdf(file="riskScore.pdf",width = 10,height = 4)

plot(line,

type="p",

pch=20,

xlab="Patients (increasing risk socre)",

ylab="Risk score",

col=c(rep("green",lowLength),

rep("red",highLength)))

abline(h=median(rt$riskScore),v=lowLength,lty=2)

legend("topleft", c("High risk", "low Risk"),bty="n",pch=19,col=c("red","green"),cex=1.2)

dev.off()

color=as.vector(rt$fustat)

color[color==1]="red"

color[color==0]="green"

pdf(file="survStat.pdf",width = 10,height = 4)

plot(rt$futime,

pch=19,

xlab="Patients (increasing risk socre)",

ylab="Survival time (years)",

col=color)

legend("topleft", c("Dead", "Alive"),bty="n",pch=19,col=c("red","green"),cex=1.2)

abline(v=lowLength,lty=2)

dev.off()

rt1=rt[c(3:(ncol(rt)-2))]

rt1=t(rt1)

annotation=data.frame(type=rt[,ncol(rt)])

rownames(annotation)=rownames(rt)

pdf(file="heatmap.pdf",width = 10,height = 4)

pheatmap(rt1,

annotation=annotation,

cluster_cols = FALSE,

fontsize_row=11,

show_colnames = F,

fontsize_col=3,

color = colorRampPalette(c("green", "black", "red"))(50) )

dev.off()

**(9) Time ROC**

library(survival)

library(survivalROC)

setwd("C:\\Users\\Administrator\\Desktop\\hcczisinew\\20 TimeROC")

rt=read.table("risk.txt",header=T,sep="\t",check.names=F,row.names=1)

pdf("ROC.pdf")

fit1<-survivalROC.C(Stime=rt$time,

status=rt$status,

marker = rt$RiskScore,

predict.time=1)

plot(fit1$FP,fit1$TP,type="l",

xlim=c(0,1),ylim=c(0,1),

xlab="1-Specificity",ylab="Sensitivity",main="Time-dependent ROC curve",col="blue",lwd=3)

abline(0,1,col="skyblue")

fit3<-survivalROC.C(Stime=rt$time,

status=rt$status,

marker = rt$RiskScore,

predict.time=3)

lines(fit3$FP,fit3$TP,type="l",

xlim=c(0,1),ylim=c(0,1),col="red",lwd=3)

fit5<-survivalROC.C(Stime=rt$time,

status=rt$status,

marker = rt$RiskScore,

predict.time=5)

lines(fit5$FP,fit5$TP,type="l",

xlim=c(0,1),ylim=c(0,1),col="green",lwd=3)

legend("bottomright",c(paste("AUC at 1 years:",round(fit1$AUC,3)),

paste("AUC at 3 years:",round(fit3$AUC,3)),

paste("AUC at 5 years:",round(fit5$AUC,3))),

lwd=4,col=c("blue","red","green"))

dev.off()

**(10) Survival curve analysis**

setwd("C:\\Users\\Administrator\\Desktop\\autophagy\\21.survival")

library(survival)

library("survminer")

rt=read.table("allrisk.txt",header=T,sep="\t")

diff=survdiff(Surv(futime, fustat) ~risk,data = rt)

pValue=1-pchisq(diff$chisq,df=1)

pValue=signif(pValue,4)

pValue=format(pValue, scientific = TRUE)

fit <- survfit(Surv(futime, fustat) ~ risk, data = rt)

pdf(file="survival.pdf",onefile = FALSE,

width = 6,

height =5)

ggsurvplot(fit,

data=rt,

conf.int=TRUE,

pval=paste0("p=",pValue),

pval.size=4,

risk.table=TRUE,

legend.labs=c("High risk", "Low risk"),

legend.title="Risk",

xlab="Time(years)",

break.time.by = 1,

risk.table.title="",

palette=c("red", "blue"),

risk.table.height=.25)

dev.off()

summary(fit)
